# Use of Cellular-Enabled Remote Patient Monitoring Device for Hypertension Management in Pregnant Women: A Feasibility Study

**DOI:** 10.1007/s10995-023-03628-1

**Published:** 2023-03-14

**Authors:** Rebecca D. Jones, M. Kathryn Allison, Heather Moody, Cheng Peng, Hari Eswaran

**Affiliations:** University of Arkansas for Medical Sciences, 4301 West Markham Street Little Rock, Little Rock, AR 72223 USA

**Keywords:** Remote patient monitoring, Cellular-enabled remote patient monitoring device, Hypertension, Pregnancy, Pregnant women

## Abstract

**Introduction:**

Hypertension affects 5–10% of pregnancies in the United States. Chronic hypertension during pregnancy can have a significant impact on maternal and neonatal outcomes, especially in rural populations. Pregnancies complicated by hypertension are currently managed through frequent clinic visits or extended hospital stays. Cellular-enabled remote patient monitoring devices provide an alternative treatment method for women in rural areas.

**Research Aim:**

This study aimed to measure the feasibility of and patient satisfaction with using an integrated model of cellular-enabled remote patient monitoring devices for blood pressure supported by a 24/7 nurse call center.

**Methods:**

In a mixed methods pilot study, twelve women with chronic hypertension during pregnancy were given cellular-enabled BodyTrace^™^ blood pressure cuffs and weight scales. Participants’ blood pressures were continuously monitored by a nurse call center. Participants completed a survey and a brief semi-structured interview after two weeks.

**Results:**

Participants scored low on stress and anxiety with mean scores of 5.45 (SD = 3.56) and 8.09 (SD 3.62), respectively. Participants scored high on behavioral intention, system usability, and perceived benefits with mean scores of 8.73 (SD = 2.53), 75.91 (SD = 23.70), and 19.64 (SD = 5.92), respectively. Participants perceived benefits to using the device, including increased monitoring by health professionals, increased self-awareness, decreased number of clinic visits, and convenience of use. Perceived disadvantages included higher readings when compared to clinical readings.

**Discussion:**

Cellular-enabled remote patient monitoring devices for blood pressure are a valuable tool for managing treatment of pregnancies complicated by hypertension.

## Significance

Previous research on remote patient monitoring (RPM) for hypertension management in postpartum and pregnant women found high participant satisfaction, decrease in blood pressure (BP) rates, increased BP management, and overall positive perceptions. This is the first study to assess the feasibility and acceptability of cellular-enabled RPM BP devices, which do not require a smartphone or app, combined with a 24/7 nurse call center with continuous BP monitoring and response to high readings for better care management. This mixed-method study explored patient satisfaction with the integrated BP system and perceived advantages and disadvantages of the device.

## Introduction

Healthcare disparities are prevalent in obstetrical care in the United States (US), impacting more than 27 million women and infants and leading to significant resource and financial burdens for rural states (Rural Health Information Hub, 2019). Academic health science centers typically provide the only referral source for high-risk pregnancies in rural states, and many of these women travel several hours for prenatal visits (Rhoads, 2017). In addition to financial and geographical burdens, these women experience treatment delays which further compromise their health outcomes. Telecommunication technologies, such as remote patient monitoring (RPM), can support and promote long-distance clinical health care by enhancing quality of care, reducing costly readmissions and emergency department visits, and keeping costs manageable for small, rural hospitals (Lustig, 2012).

Management of chronic conditions during pregnancy, such as hypertension, can have a significant impact on optimal maternal and neonatal outcomes, especially in rural populations (Hansen & Moloney, 2020; Fingar et al., 2018). Hypertension during pregnancy, a common indicator of preeclampsia, is a major health concern for women and their newborns—not only in the US, but worldwide (Magro-Malossa et al., 2017, Magee et al., 2014, Hypertension in Pregnancy, 2013). The incidence of hypertension in pregnancy has increased by 25% in the past two decades, currently affecting 5–10% of all pregnancies in the US (Ananth et al., 2013, Lo, Mission, & Caughey, 2013). This has disproportionately affected minority women and women living in rural areas (Howell et al., 2017; Lisonkova et al., 2016, & Creanga et al., 2014). Both in Arkansas and nationally, maternal death rates are higher for racial minorities and socioeconomically disadvantaged populations (Lanssens et al., 2018).

Early identification of hypertension or preeclampsia can help minimize hospitalizations, reduce morbidity and long-term healthcare costs, and improve maternal outcomes. The current standard of care for management of pregnancies complicated by hypertension is close monitoring through frequent clinic visits or hospitalization until delivery. However, RPM offers an alternative solution for patient management in rural, low-health resource settings. The use of cellular-enabled blood pressure (BP) devices can be an effective tool in providing physicians the physiological data necessary to more accurately track and respond to patient’s BP readings thus allowing a reduction in the number of clinic visits for patients, especially those living in rural areas where frequent trips to the doctor’s office may not be feasible. Instant access to data also allows physicians the ability to address concerns between patient visits, leading to better health outcomes for both the woman and infant. The ability to monitor BP readings for pregnant women with a history of hypertension or preeclampsia is imperative for improved health outcomes.

### Purpose

This study aimed to measure patients’ satisfaction with and feasibility of using an integrated model of cellular-enabled RPM devices for BP supported by a 24/7 nurse call center.

## Methods

### Study Design

This study was a mixed methods pilot study utilizing a survey and brief semi-structured qualitative interview to assess the feasibility and acceptability of RPM BP devices supported by a nurse call center for pregnancies complicated by hypertension. Twelve women identified as having a history of chronic hypertension were invited to participate in this study. Participants were given a cellular-enabled BodyTrace^™^ kit that included a BP cuff and weight scale for home use according to their healthcare providers’ recommendation. While the weight scale was provided as part of a kit from BodyTrace^™^, this paper is solely focused on the BP monitors, and weight data was not analysed. While most RPM devices require the use of Wi-Fi or Bluetooth to operate, the BodyTrace^™^ BP device is equipped with built-in cellular transmission capabilities using cell towers to transmit participant’s BP readings directly to the associated website that their healthcare providers can then access in real-time. Due to the nature of these devices, no app or Wi-Fi is needed to use the device, creating a greater level of ease and access for participants. BP parameters were set according to the University of Arkansas for Medical Sciences (UAMS) High Risk Pregnancy Program (HRPP) guidelines and were closely monitored by registered nurses in the Nurse Call Center (NCC), which is staffed 24 hours a day, 7 days a week. If a participant’s BP readings were outside of the set range, NCC staff would contact the patient to triage and provide further instructions as needed. Subjects were asked to monitor their BP and weight for two weeks after which they were invited to complete a semi-structured interview and survey. The study was approved by the UAMS Institutional Review Board on December 3rd, 2020 (#261,908).

### Participants

Participants were women whose pregnancy was complicated with hypertension. Eligibility criteria included (a) pregnant females in their second trimester between18-50 years of age whose prenatal care was received at the [institution blinded for peer review] Women’s Health Clinic and who (b) had elevated BP meeting the criteria of systolic ≥ 140 and/or diastolic ≥ 90 for at least one reading.

Twelve women who met the eligibility criteria were consented to participate; however, one participant was lost to follow-up, resulting in a total of 11 participants included in the final analysis. Participants received a $20 gift card as compensation after completing the interview and survey.

### Data Collection

#### Quantitative Data

Participants completed the web-based, self-administered survey after they had used the RPM BP device for two weeks. Informed consent was obtained verbally prior to enrollment. The survey contained six sections including (a) demographic characteristics, (b) perceived stress, (c) anxiety, (d) behavioral intention, (e) system usability and satisfaction, and (f) perceived benefits. The survey took approximately 10–15 min to complete.

#### Qualitative Data

Of the 12 pregnant patients enrolled in the study, 11 were interviewed after they had used the RPM BP device for two weeks. A trained research associate used a semi-structured interview guide to facilitate the interview over the phone. The interviews lasted an average of 5–10 min. All interviews were audio-recorded and transcribed verbatim. Participant confidentiality was maintained by assigning all participants a unique identifying code on all study materials.

### Measurements

#### Perceived Stress

Perceived stress was assessed using Cohen’s Short Form Perceived Stress Scale (PSS-4)—a brief, four-item self-report instrument using a five-point Likert scale (Cohen et al., 1983). The calculated Cronbach’s Alpha for the PSS-4 was 0.87.

#### Anxiety

The Patient-Reported Outcomes Measurement Information System (PROMIS) Anxiety Short Form, a four-item, validated self-report instrument that uses a five-point Likert scale was used to measure anxiety (Northwestern University’s Feinberg School of Medicine). The PROMIS Anxiety Short Form has a calculated Cronbach’s Alpha of 0.84 with higher sum composite scores correlating to higher levels of perceived anxiety.

#### Behavioral Intention

Behavioral intention was assessed using two questions: “If the opportunity presented itself again, I would use the system to monitor my health from home” and “I would recommend the system to other women eligible to monitor their health from their home”. The two questions used a five-point Likert scale. The calculated Cronbach’s Alpha was 0.98.

#### System Usability and Satisfaction

System usability and satisfaction was assessed using a 10-item validated instrument to assess participant satisfaction with the usability of the device (Brooke, 1996). The calculated Cronbach’s Alpha was 0.88.

#### Perceived Benefits

Participant perceived benefits associated with the use of the device was assessed using a five-item Likert-scale instrument adapted from previous research on mobile health devices (Rhoads et al., 2017). The calculated Cronbach’s Alpha was 0.95.

### Data Analysis

#### Quantitative Data

For the quantitative results, descriptive statistics of participant’s demographic characteristics were reported in Table 1. The mean score and standard deviation were reported for each scale in Table 2. Cronbach’s alphas were calculated for each scale.

**Table 1:** Characteristics of patients (*N* = 11)

Characteristic			n (%)		Total n Responded
Race					11
Black or African American			3 (27.27%)		
White			8 (72.73%)		
Hispanic					9
No			9 (100.00%)		
Yes			0 (0.00%)		
Marital Status					11
Married			7 (63.64%)		
Divorced, separated, or widowed			2 (18.18%)		
Others			2 (18.18%)		
Highest Education					11
9th grade to 12th grade			1 (9.09%)		
High school graduate or GED			3 (27.27%)		
Some college or technical school			5 (45.45%)		
College graduate or higher			2 (18.18%)		
Annual Household Income					11
< $15,000			2 (18.18%)		
$15,000 to < $20,000			2 (18.18%)		
$20,000 to < $25,000			0 (0.00%)		
$25,000 to < $35,000			3 (27.27%)		
$35,000 to < $50,000			3 (27.27%)		
$50,000 to < $75,000			1 (9.09%)		
>= $75,000			0 (0.00%)		
Employment					11
Full-time			2 (18.18%)		
Part-time			2 (18.18%)		
Unemployed			6 (54.55%)		
Unemployed Disabled		1 (9.09%)			
Number of Children aged < 18					11
0		2 (18.18%)			
1		5 (45.45%)			
2		2 (18.18%)			
3		1 (9.09%)			
4		1 (9.09%)			
First Pregnancy					11
No		8 (72.73%)			
Yes		3 (27.27%)			
Last Baby Delivered					8
Pre-term		5 (62.50%)			
Term		3 (37.50%)			
Post-term		0 (0.00%)			
Last Baby Birth Weight Range					8
3 to 4 pounds		3 (37.50%)			
6 pounds or more	5 (62.50%)				
First pregnancy or postpartum period with blood pressure issues or preeclampsia				8	
No	1 (12.50%)				
Yes	7 (87.50%)				

**Table 2:** Mean, standard deviation for measures

Measures	N	M	SD
Perceived Stress	11	5.45	3.56
Anxiety	11	8.09	3.62
Behavioral Intention	11	8.73	2.53
System Usability and Satisfaction	11	75.91	23.70
Perceived Benefits	11	19.64	5.92

#### Qualitative Data

Using MAXQDA Plus 20 qualitative analysis software (VERBI Software, Belin, Germany), two researchers analyzed the qualitative data using thematic analysis to identify major themes. First, all interview transcripts were read by the coders to familiarize themselves with the data. The codebook was then developed in an iterative process of discussion and refinement. Coders used constant comparative analysis to search line by line for patterns, codes, and themes. As new codes and themes emerged, the coders reviewed previous interviews to ensure consistency. After all transcripts were coded, the data analysis team identified major themes and exemplary quotations.

## Results

### Characteristics of the Sample

Sociodemographic characteristics of the 11 participants are reported in Table 1. Most participants were white (72.7%), non-Hispanic (100%), married (63.6%), had attended at least some college or technical school (63.6%), and reported an annual household income between $25,000 – $75,000 (63.6%). One out of four (27%) participants reported that this was their first pregnancy. Most participants had one or more children (81.8%). The majority of participants had previously delivered pre-term (62.50%) and reported this to be their first pregnancy with BP issues or preeclampsia (87.50%).

### Quantitative Results

Participants’ mean scores and standard deviations for each scale were reported in Table 2. For perceived stress (PSS-4), participants received a score ranging from 0 to 16, with higher scores indicating higher levels of perceived stress. The mean score for the PSS-4 was 5.45 with a standard deviation of 3.56, indicating relatively low levels of perceived stress among participants. Anxiety was assessed using the PROMIS Anxiety Short Form, which scores from 4 to 20. The average anxiety score was 8.09 (SD = 3.62). Behavioral intention was assessed using two Likert-scale questions, with participants receiving a score between 2 and 10. The average behavioral intention score was 8.73 (SD = 2.53), indicating high intention by participants to continue using the device. The System Usability and Satisfaction scale produces a composite score between 0 and 100, with higher scores indicating higher satisfaction with the device. The average score was 75.91 (SD = 23.70), which suggests high participant satisfaction with the device. Lastly, perceived benefits were assessed using five Likert-scale questions, with a composite sum score between 5 and 25. The average perceived benefit score was 19.64 (SD = 5.92), indicating that participants associated high benefits with using the device.

### Qualitative Results

#### Advantages

Participants described the advantages of using the RPM BP device, including (1) perceptions that their care was better, (2) BP being remotely monitored by a health professional rather than exclusively by the patients themselves, (3) increased participant empowerment, (4) convenience, and (5) ease of use of the device.

##### Perceived Better Care

 Most participants felt that their care was better than patients who did not use the device because the device alerts health professionals to high BP readings. Health professionals then respond to the patient and recommend next steps in their care. One participant felt that she potentially had better outcomes because she used the device, saying, “*Because I took my blood pressure with it, and it notified the nurse, they sent me to triage, and I ended up having my baby early because I had preeclampsia that hadn’t been noticed yet. So yeah, we were able to catch that happening before it got too out of hand*.”

##### Remote Monitoring by a Health Professional

Participants thought one advantage of using the device was that their readings were remotely monitored by a health professional, who would be notified if a reading was high. They liked this because it placed the responsibility of responding to a high reading on a trained professional rather than the patients themselves. Several participants liked their BP being remotely monitored because they either would not have taken their BP otherwise or did not feel confident interpreting their own results. One participant said, “*I definitely wouldn’t have taken it on my own, probably, and if I did and had something high, I’m like pretty ignorant on blood pressure—like what’s normal or what they would want—so probably, if I would have taken it, I wouldn’t have done anything with that information*.” Another said, “*Since it, you know, sends a transmission, I guess that’s good thing ‘cause I would have ignored stuff like that high reading*.”

##### Participant Empowerment

Participants felt that they were better able to care for themselves and their health because they were aware of their BP when using the device. One participant said, “*I feel like it just made me more aware and conscious of the things that I was allowing to stress me out*.” Other participants said, “*I had an opportunity to keep an eye on my health*,” and “*Since I was pregnant at the time and was experiencing high blood pressure, it was good to keep a very watchful eye on it*.”

##### Convenient

Participants liked that they could use the device anywhere because it is cellular-enabled and does not have to be connected to the internet to transmit readings. One participant said, “*I was able to take it everywhere with me*.” Participants also liked that they did not have to go into a clinic or hospital to have their BP read, and instead, could do it from home. One participant said, “*I mean, if it wasn’t for the reading being able to be sent directly to ya’ll, I would have to come into [city name] probably quite more often during the week instead of just sitting at home and relaxing*.” Some liked that they did not have to log their readings. One said that she liked “*the fact that it can get sent directly to the doctor’s office versus me having to remember or write it down or something*.”

##### Easy to Use

Many participants described the device as “*easy to use*” or “*self-explanatory*.” One participant said, “*It was as simple… you know, you put the cuff on and hit the button, and it was just really easy. It wasn’t complicated.”* Another participant said, “*I felt very comfortable using it.”* One participant liked that it is “*just a standard blood pressure cuff”* and not different from devices that she had used in the past.

### Disadvantages

While some participants reported no issues, others reported concerns with the device itself, such as problems with wearing the device or perceiving that it gave higher readings than clinical BP monitors. Others had concerns with the remote monitoring process, such as the alerts and calls from health professionals when readings were high.

#### Issues with the Device

When asked about disadvantages of the device, some participants reflected on the accuracy of the device and perceived that readings were too high. One participant said, “*It wasn’t as accurate. Again, it was the readings were a little bit higher, and it was pretty frustrating. I noticed that when I would go to the doctor’s office, numbers would be a little lower than what it would show on that [remote] blood pressure [monitor].”* Few participants reflected on their experience wearing the BP cuff, although one participant stated, “*The cuff itself—because it was so small, so it was very uncomfortable, and it got really, really tight. So, I didn’t really care for it to be honest.*” Another said that she had issues figuring out how to position the cuff appropriately on her arm, saying, “*the only thing [that was] kind of a bit annoying was trying to get the cuff adjust[ed] on my arm right so it would read correctly, but I mean, once I figured out, you know, how it needed to be positioned, it wasn’t a big deal. That was the only issue I had with it*.”

#### Issues with the Response Protocol

Because the program protocol required a phone call from a health professional each time that a participant received a high BP reading, several participants found the response overwhelming, receiving either many calls or believing that their readings were inaccurate and did not warrant a response from a health professional. One participant described these calls as stressful, saying, “*Well, this is, I’m sure, not everyone is the same, but I freak out really easy so, whenever I have, I guess, a medical professional call me and kind of urgently be like, ‘you need to go to triage’ or something—that kind of freaked me out. I don’t wanna say it made it worse but maybe like momentarily. I mean, it was stressful, but also it was, you know, obviously something that needed to be monitored and taken care of if it did get high, which it did.”* Participants said they received these calls even when a high reading was immediately followed by a normal reading. One participant said, “*Apparently if I had a high reading, even if I took it like 2 minutes later, they would call a bunch of times*.”

## Discussion

This pilot study explored the feasibility and acceptability of using cellular-enabled RPM devices supported by a 24/7 nurse call center to monitor BP in pregnancies complicated by hypertension. The study aimed to measure participants’ satisfaction with the device, their perceived stress, anxiety, behavioral intentions to continue using the device, and perceived benefits associated with using the device. Further perceived advantages and disadvantages associated with the RPM BP device were explored in a semi-structured interview. Previous research on RPM for hypertension has focused on postpartum women (Payakachat et al., 2020; Hoppe et al., 2019) or used Bluetooth devices that require a smartphone and app (Ganapathy et al., 2016; Marko et al., 2016). In a small pilot study that aimed to assess the feasibility of using Bluetooth RPM BP devices for prenatal care, it was found that patients demonstrated high satisfaction with the device (Marko et al., 2016). A similar study in which pregnant participants were provided a Bluetooth RPM BP device found that 90% of the women agreed that the device was easy to use and 78% indicated they would prefer testing at home (Ganapathy et al., 2016). To our knowledge, the current study is the first to assess the use of a cellular-enabled RPM BP device supported by a 24/7 nurse call center for hypertension in pregnancy.

Previous studies on RPM for hypertension have found high participant satisfaction with using the devices (Payakachat et al., 2020; Hoppe et al., 2019; Marko et al., 2016) and have shown significant decrease in BP (Logan et al., 2007). One previous study on postpartum women found that the majority of the participants preferred telehealth to in-clinic care and viewed telehealth as a secure and easy monitoring technique (Hoppe et al., 2019). Another study found overall positive perceptions of RPM devices due to decreased travel time to clinics and empowerment of participants to manage their own health (Payakachat et al., 2020). These previous findings align with the results of this study with convenience and ease of use being cited by many participants as advantages to using the device. In addition, participants in the current study stated that having their BP readings continuously monitored by a health professional was an advantage of using the device as it removed the pressure of responding appropriately to high BP readings from the participant. Many participants perceived that they received better care and BP management, including increased self-awareness of their own BP readings, as a result of using the RPM device.

Disadvantages associated with using RPM devices include variability in BP readings when compared to clinic readings. A previous study in the UK in which participants were provided RPM BP devices and asked to record their BP readings in an app, found that at-home readings were consistently lower than clinical readings (Kalafat et al., 2018) which was similar to adult non-pregnant reports. On the other hand, as reported by Bello et al. (2018a), two pregnancy-based studies (Naef et al. 1998, Tucker et al., 2017) found similar clinical and home measurements, while another one showed (Lo et al., 2002) a higher trend in home measurements. In our current study, some of the participants BP readings found by the RPM device were consistently higher than those found in clinic. These findings indicate that some variability to BP readings by RPM devices when compared to clinical readings is common, which is also reflected in the perceived disadvantage by some of the women in our study. While validation studies have been conducted on many at-home BP devices (Bello, 2018b), studies validating the BodyTrace^™^ RPM BP device have yet to be conducted.

There are limitations in the present study. This was a pilot study aimed at assessing the feasibility and patient acceptability of RPM BP devices. The preferred sample size for pilot studies is twelve (Julious, 2005); however, as one participant was lost to follow-up, only eleven participants were included in the final analysis. Despite the small sample size, the information found is beneficial for future studies assessing cellular-enabled RPM for pregnant women with hypertension. In addition, as the main study outcomes were self-reported, social desirability bias may have impacted both quantitative and qualitative data.

The researchers plan to expand upon these finding through implementation of a second phase in which 38 participants will be recruited to use the cellular-enabled BodyTrace^™^ BP device. In this phase, participants will use the device for 8-weeks and complete a pre-survey, post-survey, and semi-structured interview. We aim to assess adherence of and satisfaction with the integrated RPM BP system and explore associations between use of the RPM BP device and maternal outcomes. Maternal outcomes will be assessed through number of hospital admissions, hospital length of stay, and number of emergent visits during pregnancy.

## Conclusion

The use of cellular-enabled RPM BP devices supported by a 24/7 nurse call center is a valuable tool for managing treatment of women whose pregnancies are complicated by hypertension. Participants scored high on behavioral intention to continue use of the device, satisfaction with the device, and perceived benefits associated with using the device. In addition, participants reported many benefits to using the RPM BP device including increased monitoring of their BP by a health professional, increased self-awareness, decreased number of clinic visits, convenience, and ease of use. The main disadvantage mentioned by participants was higher BP readings by the RPM BP device when compared to clinical BP readings. Due to the overall positive feedback and the ability to decrease barriers to healthcare for women living in rural areas, RPM BP devices should be considered for use in managing hypertensive pregnant women in rural areas.

## Data Availability

Data and material is available.
